# The immunity-related GTPase Irga6 dimerizes in a parallel head-to-head fashion

**DOI:** 10.1186/s12915-016-0236-7

**Published:** 2016-03-02

**Authors:** Kathrin Schulte, Nikolaus Pawlowski, Katja Faelber, Chris Fröhlich, Jonathan Howard, Oliver Daumke

**Affiliations:** Max-Delbrueck-Centrum for Molecular Medicine, Crystallography, Robert-Rössle-Strasse 10, 13125 Berlin, Germany; Institute for Genetics, Department of Cell Genetics, University of Cologne, Zülpicher Strasse 47a, 50674 Cologne, Germany; Fundação Calouste Gulbenkian, Instituto Gulbenkian de Ciência, 2781-156 Oeiras, Portugal; Present address: Bayer Pharma AG, Global Biologics Research, Nattermannallee 1, 50829 Cologne, Germany

**Keywords:** Innate immunity, IRG proteins, GTPase, Dynamin superfamily, Dimerization, Oligomerization

## Abstract

**Background:**

The immunity-related GTPases (IRGs) constitute a powerful cell-autonomous resistance system against several intracellular pathogens. Irga6 is a dynamin-like protein that oligomerizes at the parasitophorous vacuolar membrane (PVM) of *Toxoplasma gondii* leading to its vesiculation. Based on a previous biochemical analysis, it has been proposed that the GTPase domains of Irga6 dimerize in an antiparallel fashion during oligomerization.

**Results:**

We determined the crystal structure of an oligomerization-impaired Irga6 mutant bound to a non-hydrolyzable GTP analog. Contrary to the previous model, the structure shows that the GTPase domains dimerize in a parallel fashion. The nucleotides in the center of the interface participate in dimerization by forming symmetric contacts with each other and with the switch I region of the opposing Irga6 molecule. The latter contact appears to activate GTP hydrolysis by stabilizing the position of the catalytic glutamate 106 in switch I close to the active site. Further dimerization contacts involve switch II, the G4 helix and the *trans* stabilizing loop.

**Conclusions:**

The Irga6 structure features a parallel GTPase domain dimer, which appears to be a unifying feature of all dynamin and septin superfamily members. This study contributes important insights into the assembly and catalytic mechanisms of IRG proteins as prerequisite to understand their anti-microbial action.

**Electronic supplementary material:**

The online version of this article (doi:10.1186/s12915-016-0236-7) contains supplementary material, which is available to authorized users.

## Background

Immunity-related GTPases (IRGs) comprise a family of dynamin-related cell-autonomous resistance proteins targeting intracellular pathogens, such as *Mycobacterium tuberculosis* [1], *Mycobacterium avium* [2], *Listeria monocytogenes* [3], *Trypanosoma cruzi* [4], and *Toxoplasma gondii* [3, 5–11]. In mice, the 23 IRG members are induced by interferons, whereas the single human homologue is constitutively expressed in some tissues, especially in testis [12]. In non-infected cells, most IRGs are largely cytosolic. However, members of a small sub-family with regulatory function [11] associate with specific intracellular membranes, with one member favoring the endoplasmic reticulum [13, 14] and others the Golgi membrane [7, 14] and the endolysosomal system [15]. Infection by certain intracellular pathogens initiates the redistribution of several effector members to the parasitophorous vacuole, followed by its disruption [7, 14, 16, 17]. In this way, IRGs contribute to the release of the pathogen into the cytoplasm and its subsequent destruction.

Irga6, one of the effector IRG proteins, localizes to the intact parasitophorous vacuole membrane (PVM) and, after disruption of the PVM, is found associated with vesicular accumulations, presumably derived from the PVM [7, 15, 18, 19]. A myristoylation site at Gly2 is necessary for the recruitment to the PVM but not for the weak constitutive binding to the ER membrane [14, 20]. An internally oriented antibody epitope on helix A between positions 20 and 24 was demonstrated to be accessible in the GTP-, but not in the GDP-bound state [20, 21]. This indicates large-scale structural changes upon GTP binding that probably include exposure of the myristoyl group, enhancing binding to the PVM. Biochemical studies indicated that Irga6 hydrolyses GTP in a cooperative manner and forms GTP-dependent oligomers in vitro and in vivo [20, 22].

Crystal structures of Irga6 in various nucleotide-loaded states revealed the basic architecture of IRG proteins, including a GTPase domain and a composite helical domain [23]. These studies additionally showed a dimerization interface in the nucleotide-free protein as well as in all nucleotide-bound states. It involves a GTPase domain surface, which is located at the opposite side of the nucleotide, and an interface in the helical domain, with a water-filled gap between the two contact surfaces. Mutagenesis of the contact surfaces suggests that this "backside" interface is not required for GTP-dependent oligomerization or cooperative hydrolysis, despite an earlier suggestion to the contrary [23].

Extensive biochemical studies suggested that GTP-induced oligomerization of Irga6 requires an interface in the GTPase domain across the nucleotide-binding site [24]. Recent structural studies indicated that a 'G interface' is typical of dynamin superfamily members, such as dynamin [25, 26], MxA [27, 28], the guanylate binding protein-1 (GBP-1) [29], atlastin [30, 31] and the bacterial dynamin-like proteins (BDLP) [32, 33]. For several of these proteins, formation of the G interface was shown to trigger GTP hydrolysis by inducing rearrangements of catalytic residues *in cis*. In dynamin, the G interface includes residues in the phosphate binding loop, the two switch regions, the '*trans* stabilizing loop' and the 'G4 loop'. For Irga6, it was demonstrated that besides residues in the switch I and switch II regions, the 3'-OH group of the ribose participates in this interface [24]. Since the signal recognition particle GTPase and its homologous receptor (called FfH and FtsY in bacteria) also employ the 3'-OH ribose group to dimerize in an anti-parallel orientation therefore activating its GTPase [34], an analogous dimerization model was proposed for Irga6 [24]. However, the crystal structure of Irga6 in the presence of the non-hydrolyzable GTP analogue 5'-guanylyl imidodiphosphate (GMPPNP) showed only subtle differences relative to the apo or GDP-bound protein and did not reveal a new dimer interface associated with the GTPase domain [23]. This structure was obtained by soaking GMPPNP in nucleotide-free crystals of Irga6, an approach which may have interfered with nucleotide-induced domain rearrangements.

To clarify the dimerization mode via the G interface, we determined the GMPPNP-bound crystal structure of a non-oligomerizing Irga6 variant. The structure revealed that Irga6 can dimerize via the G interface in a parallel head-to-head fashion. This dimerization mode explains previously published biochemical data, and shows in particular how the 3'-OH group of the ribose participates in the assembly. Our data suggest that a parallel dimerization mode may be a unifying feature in all dynamin and septin superfamily proteins.

## Results

Previous results indicated that Irga6 mutations in a loosely defined surface region (the "secondary patch"), which is distant from the G-interface and only slightly overlapping with the backside interface (see below), individually reduced GTP-dependent oligomerization [24]. A combination of four of these mutations (R31E, K32E, K176E, and K246E) essentially eliminated GTP-dependent assembly (Additional file 1: Figure S1) and allowed crystallization of Irga6 in the presence of GMPPNP. Crystals diffracted to 3.2 Å resolution and displayed one exceptionally long unit cell axis of 1289 Å (Additional file 1: Table S1). The structure was solved by molecular replacement and refined to R_work_/R_free_ of 29.7 %/31.7 % (Additional file 1: Table S2). The asymmetric unit contained seven Irga6 molecules that were arranged in a helical pattern along the long cell axis (Additional file 1: Figure S2).

**Fig. 1:**
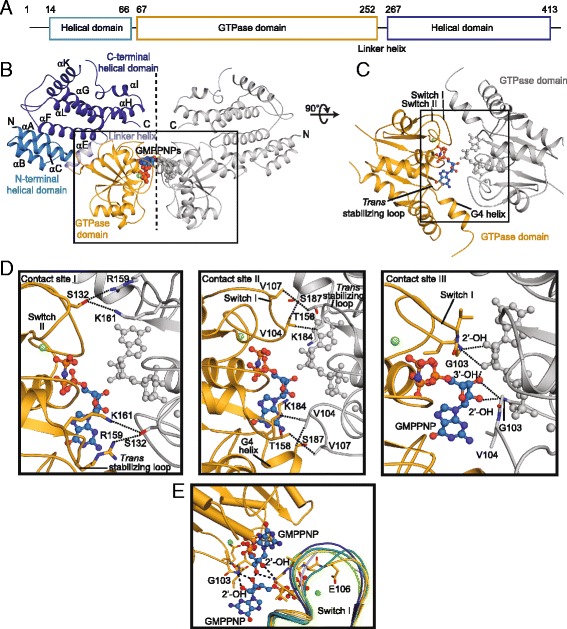
Structure of the Irga6 dimer. **a** Schematic view of the domain architecture of mouse Irga6. The first and last amino acids of each domain are indicated. **b** Ribbon-type representation of the Irga6 dimer. In the *left* molecule, domains are colored according to the domain architecture, the *right* molecule is colored in grey. The nucleotide and Mg^2+^ ion (green) are shown in *sphere* representation. The GTPase domain dimer is *boxed*. The *dotted line* indicates a 2-fold axis. Secondary structure was numbered according to ref. [23]. **c** *Top view* on the GTPase domain dimer. **d** Magnification of the contact sites. *Dotted lines* indicate interactions. **e** Superposition of different switch I conformations in the asymmetric unit; the same colors as in Additional file 1: Figure S2 are used for the switch I regions of the individual subunits. Switch I residues of subunit A (yellow) involved in ribose binding are labelled and shown in stick representation. *Irga6* immunity-related GTPase 6

Like other dynamin superfamily members, the GTPase domain of Irga6 comprises a canonical GTPase domain fold, with a central β-sheet surrounded by helices on both sides (Fig. 1a-c). The helical domain is a bipartite structure composed of helices αA-C at the N-terminus and helix αF-L at the C-terminus of the GTPase domain. Overall, the seven molecules in the asymmetric unit are very similar to each other, with root mean square deviations (rmsd) ranging from 0.32 – 0.45 Å over all Cα atoms. The structures of the seven molecules also agree well with the previously determined structure of native GMPPNP-bound Irga6 (PDB: 1TQ6; rmsd of 1.00-1.13 Å over all Cα atoms).

The seven Irga6 molecules in the asymmetric unit form various higher order contacts in the crystals. Within the asymmetric unit, six molecules dimerize via the symmetric backside dimer interface (buried surface area 930 Å^2^), and the remaining seventh molecule forms the same type of interaction with its symmetry mate of the adjacent asymmetric unit (Additional file 1: Figure S2a, b, Figure S3). This indicates that the introduced mutations in the secondary patch, from which only Lys176 is part of the backside interface, do, in fact, not prevent this interaction.

Another assembly interface with a buried surface area of 450 Å^2^, which we call the “tertiary patch”, was formed via two interaction sites in the helical domains (Additional file 1: Figure S2c, d, S3). In this interface, helices αK from two adjacent molecules form a hydrogen bonding network involving residues 373-376. Furthermore, two adjacent helices αA form hydrophobic contacts. It was previously shown that the double mutation L372R/A373R did not prevent GTP-induced assembly [24], so there is currently no evidence supporting an involvement of this interface in higher-order oligomerization.

Strikingly, molecule A of one asymmetric unit assembled with an equivalent molecule of the adjacent asymmetric unit via the G-interface in a symmetric parallel fashion via a 470 Å^2^ interface. This assembly results in a butterfly-shaped Irga6 dimer in which the helical domains protrude in parallel orientations (Fig. 1b, Additional file 1: Figure S3). In contrast, the other six molecules in the asymmetric unit do not assemble via the G interface.

The G interface in molecule A can be subdivided into three distinct contact sites (Fig. 1c, d). Contact site I is formed between R159 and K161 in the *trans* stabilizing loops, and S132 in the switch II regions of the opposing molecules. Contact site II features polar and hydrophobic interactions formed by switch I (V104, V107) with a helix following the guanine specificity motif (G4 helix, K184 and S187) and the *trans* stabilizing loop (T158) of the opposing GTPase domain. In contact site III, G103 of switch I interacts via its main chain nitrogen with the exocyclic 2’-OH and 3’-OH groups of the opposing ribose in *trans*, whereas the two opposing exocyclic 3’-OH group of the ribose form hydrogen bonds with each other. Via the ribose contact, switch I is pulled towards the opposing nucleotide (Fig. 1e). In turn, E106 of switch I reorients towards the nucleotide and now participates in the coordination of the Mg^2+^ ion (Fig. 1e, Additional file 1: Figure S4). E106 was previously shown to be essential for catalysis [24], and the observed interactions in contact site III explain how dimerization via the ribose is directly coupled to the activation of GTP hydrolysis.

The G interface is in full agreement with previously published biochemical data that indicate crucial roles of E77, G103, E106, S132, R159, K161, K162, D164, N191, and K196 for oligomerization and oligomerization-induced GTP hydrolysis [24]. All of these residues directly participate in contacts (G103, S132, R159, and K161) or are in direct vicinity to the interface (E77, E106, K162, D164, and N191). Residues E77, K162, and D164 appear to orient the *trans* stabilizing loop which is involved in interface formation in contact site II. In the earlier model of an anti-parallel G interface, it was not possible to position the side chain of R159 to avoid steric conflict [24]. In the present structure, the side-chain of R159 projects laterally along the G interface and, therefore, does not cause a steric conflict.

**Fig. 2:**
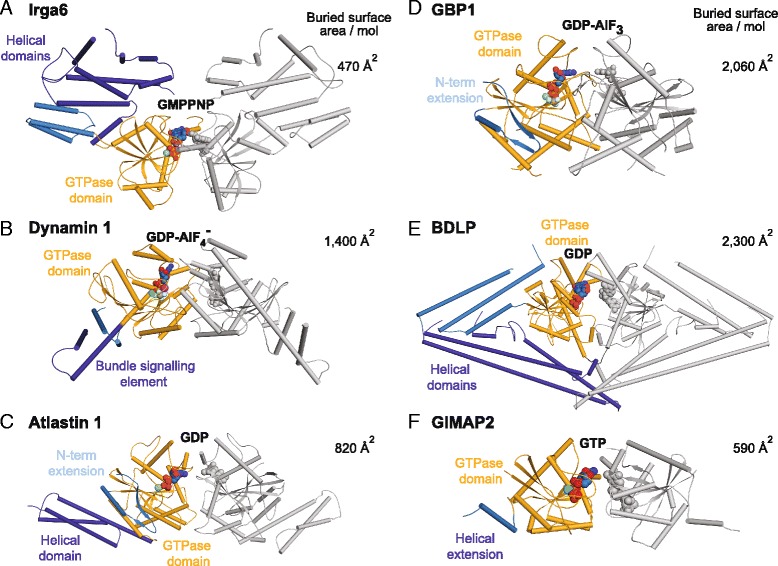
A conserved dimerization mode via the G interface in dynamin and septin GTPases. The overall architecture of the parallel GTPase domain dimer of Irga6 is related to that of other dynamin and septin superfamily proteins. The following structures are shown in cylinder representations, in similar orientations of their GTPase domains: **a** the GMPPNP-bound Irga6 dimer, **b** the GDP-AlF_4_^-^-bound dynamin 1 GTPase-minimal BSE construct [pdb 2X2E], **c** the GDP-bound atlastin 1 dimer [pdb 3Q5E], **d** the GDP-AlF_3_- bound GBP1 GTPase domain dimer [pdb 2B92], **e** the BDLP dimer bound to GDP [pdb 2J68] and **f** the GTP-bound GIMAP2 dimer [pdb 2XTN]. The GTPase domains of the *left* molecules are shown in orange, helical domains or extensions in blue. Nucleotide, Mg^2+^ (green) and AlF_4_^-^ are shown in *sphere* representation, the buried interface sizes per molecule are indicated on the *right. Irga6* immunity-related GTPase 6, *GMPPNP* 5'-guanylyl imidodiphosphate, *GTP* guanosine-triphosphate, *BDLP* bacterial dynamin like protein, *GIMAP2*, GTPase of immunity associated protein 2

The buried surface area per molecule (BSA) of the G interface in Irga6 is relatively small (470 Å^2^) compared to that of other dynamin superfamily members, such as dynamin (BSA: 1400 Å^2^), atlastin (BSA: 820 Å^2^), GBP-1 (BSA: 2060 Å^2^), BDLP (BSA: 2300 Å^2^) or the septin-related GTPase of immunity associated protein 2 (GIMAP2) (BSA: 590 Å^2^) (Fig. 2). However, the relative orientations of the GTPase domains in these dimers are strikingly similar, and the same elements, such as switch I, switch II, the *trans* activating and G4 loops are involved in the parallel dimerization mode in all of these GTPase families.

## Discussion

IRG proteins are crucial mediators of the innate immune response in mice against a specific subset of intracellular pathogens, all of which enter the cell to form a membrane-bounded vacuole without engagement of the phagocytic machinery. As members of the dynamin superfamily, IRGs oligomerize at cellular membranes in response to GTP binding. Oligomerization and oligomerization-induced GTP hydrolysis are thought to induce membrane remodeling events ultimately leading to disruption of the PVM. Recent structural and mechanistic analyses have begun to unravel the molecular basis for the membrane-remodeling activity and mechano-chemical function of some members (reviewed in [35]). For example, for dynamin and atlastin, it was shown that GTP binding and/or hydrolysis leads to dimerization of the GTPase domains and to the reorientation of the adjacent helical domains. The resulting domain movement was suggested to act as a “power stroke” during membrane remodeling events [25]. However, for other dynamin superfamily members such as IRGs, the molecular basis for GTP hydrolysis and the exact role of the mechano-chemical function are still unclear.

Our structural analysis of an oligomerization- and GTPase-defective Irga6 mutant indicates that Irga6 dimerizes via the G interface in a parallel orientation. Only one of the seven Irga6 molecules in the asymmetric unit formed this contact pointing to a low affinity interaction via the G interface, which is in agreement with its small size. In the crystals, dimerization via the G interface is promoted by the high protein concentrations which may mimic a situation when Irga6 oligomerizes on a membrane surface. Such a low affinity interaction mode may allow reversibility of oligomerization following GTP hydrolysis. Similar low affinity G interface interactions were reported for dynamin [26] and MxA [27].

The dimerization mode is strikingly different from the previously proposed anti-parallel model [24] that was based on the crystal structure of the signal recognition particle GTPase, SRP54 and its homologous receptor [34]. However, the G dimer interface is reminiscent of the GTPase domain dimers observed for several other dynamin superfamily members, such as dynamin, GBP1, atlastin, and BDLP. It was recently shown that septin [36] and septin-related GTPases, such as the Tocs GTPases [37] or GTPases of immunity related proteins (GIMAPs) [38], also employ a GTP-dependent parallel dimerization mode. Based on phylogenetic and structural analysis, these observations suggest that dynamin and septin superfamilies are derived from a common ancestral membrane-associated GTPase that featured a GTP-dependent parallel dimerization mode [38]. Importantly, our analysis indicates that IRGs are not outliers, but bona-fide representatives of the dynamin superfamily.

Whereas the overall dimerization mode is similar in septin and dynamin GTPases, family-specific differences in the G interface and the oligomerization interfaces exist. For example, the involvement of the 2’ and 3’-OH groups of the ribose in the dimerization interface of Irga6 has not been observed for other dynamin and septin superfamily members. The surface-exposed location of the ribose in the Irga6 structure, with a wide-open nucleotide-binding pocket, facilitates its engagement in the dimerization interface. This contact, in turn, appears to activate GTP hydrolysis by inducing rearrangements in switch I and the positioning of the catalytic E106. During dimerization of GBP1, an arginine finger from the P loop reorients towards the nucleotide *in cis* to trigger GTP hydrolysis [29]. In dynamin, the corresponding serine residue coordinates a sodium ion that is crucial for GTP hydrolysis [26]. Irga6 bears Gly79 at this position, which in the dimerizing molecule A appears to approach the bridging imido group of GMPPNP via a main chain hydrogen bond. Higher resolution structures of the Irga6 dimer in the presence of a transition state analogue are required to show whether Gly79 directly participates in GTP hydrolysis or whether it may also position a catalytic cation.

In dynamin, further assembly sites are provided by the helical domains which assemble in a criss-cross fashion to form a helical filament. In dynamin-related Eps15 homology domain containing proteins (EHDs), a second assembly interface is present in the GTPase domain [39]. For Irga6, additional interfaces in the helical domain are presumably involved in oligomerization, such as the secondary patch residues whose mutation prevented oligomerization in the crystallized mutant. Further structural studies, especially electron microscopy analysis of the Irga6 oligomers, are required to clarify the assembly mode via the helical domains and to show how these interfaces cooperate with the G interface to mediate the regulated assembly on a membrane surface. Notably, we did not observe major rearrangements of the helical domain versus the GTPase domain in the Irga6 molecules that dimerized via the G interface. In a manner similar to BDLP [33], such large-scale conformational changes may be induced by membrane binding. Our structural analysis and the identification of the G-interface paves the way for determining the specific assembly of Irga6 into a membrane-associated scaffold as the prerequisite to understand its action as an anti-parasitic machine.

## Methods

### Protein expression and purification

Selenomethionine-substituted *Mus musculus* Irga6^R31E, K32E, K176E, K246E^ was expressed as a GST-fusion from the vector pGEX-4T-2 in BL21 Rosetta2(DE3) cells according to reference [40]. Protein was purified as previously described [24] and the protein stored in small aliquots at a concentration of 118 mg/mL in 50 mM Tris-HCl, pH 7.4, 5 mM MgCl_2_, 2 mM DTT.

### Biochemical analyses

Oligomerization and GTPase assays for the Irga6^R31E, K32E, K176E, K246E^ mutant were carried out as described in [24].

### Protein crystallization

The protein was gently thawed on ice and diluted to a final concentration of 10 mg/mL with buffer containing 20 mM Tris-HCl, pH 7.5, 8 mM MgCl_2_, 3 mM DTT. GMPPNP was added to a final concentration of 2 mM. Crystallization was carried out in a 96 well format using the sitting drop vapor diffusion method. The reservoir contained 100 mM HEPES-NaOH pH 7.0, 9 % PEG4000, 6 % isopropanol. The sitting drop was set up using an Art Robbins Gryphon system and consisted of 200 nL protein solution and 200 nL reservoir solution.

For cryo-protection, crystals were transferred into a cryo solution containing 33 % PEG4000, 3 % isopropanol, 50 mM HEPES pH 7.0, 4 mM MgCl_2_, 2 mM DTT, and 2 mM GMPPNP at 4 °C for at least 5 sec. Crystals were screened for diffraction at beamline BL 14.1 at BESSY II, Berlin, Germany.

### Data collection

All data were recorded at beamline P11 at PETRA III, DESY Hamburg, Germany using a PILATUS 6 M detector. To achieve spot separation along the long cell axis, three data sets were collected with a φ increment of 0.05/0.1° at a temperature of 100 K using detector distances between 1300 and 598.5 mm (Additional file 1: Table S1). The wavelength was 0.972/0.979 Å. Calculation of an optimal data collection strategy was done with the Mosflm software [41]. The high- and low-resolution datasets were processed and merged using the XDS program suite [42].

### Structure solution and refinement

Structure solution was done by molecular replacement with Phaser [43] employing the structure of Irga6 without nucleotide [PDB: 1TQ2] as search model [23]. Atomic model building was done by C*oot* [44]. Iterative refinement was done using Phenix at a maximum resolution of 3.2 Å [45]. For the refinement strategy, a seven-fold non-crystallographic symmetry as well as one molecule of Irga6 [PDB: 1TQ4] [23] as high resolution reference structure was chosen. Five percent of the measured X-ray intensities were set aside from the refinement as cross-validation [46]. Methionine sites in the protein were confirmed by the anomalous signal of the selenium atoms. Protein superposition was done with lsqkab [47] and the PyMol Molecular Graphics System, Version 1.3 Schrödinger, LLC. Figures were prepared using the PyMOL Molecular Graphics System, Version 1.7.4 Schrödinger, LLC. Evaluation of atom contacts and geometry of the atomic model was done by the Molprobity server [48]. Interface sizes were calculated by the PISA server [49].

### Accession numbers

The Irga6 coordinates were submitted to the Protein Data Bank (pdb) database with accession code 5fph. http://www.rcsb.org/pdb/explore/explore.do?structureId=5fph.

## Conclusions

Our study indicates that Irg proteins dimerize via the G interface in a parallel head-to-head fashion thereby facilitating GTPase activation. These findings contribute to a molecular understanding of the anti-parasitic action of the Irg protein family and suggest that Irgs are bona-fide members of the dynamin superfamily.

## Additional file

Additional file 1: Supplementary Material (all files combined). **Table S1.** Data collection statistics. **Table S2.** Refinement statistics. **Figure S1.** Mutations R31E, K32E, K176E, K246E eliminate GTP-dependent oligomerization and GTP hydrolysis of Irga6. **Figure S2.** Packing of Irga6 molecules in the crystal lattice. **Figure S3.** Contact surfaces in Irga6. **Figure S4.** Electron density map of the catalytic site in molecule A. (PDF 3.88 mb)

## Abbreviations

BDLP: Bacterial dynamin like protein
EHD2: Eps15 homology domain containing protein 2
GBP: Guanylate-binding protein
GDP: Guanosine-diphosphate
GIMAP2: GTPase of immunity associated protein 2
GMPPNP: 5'-guanylyl imidodiphosphate
GTP: Guanosine-triphosphate
IRG: Immunity-related GTPase
Irga6: Immunity-related GTPase 6
MxA: Myxovirus resistance protein A
PVM: Parasitophorous vacuolar membrane
