# Supplementary material for: The immunity-related GTPase Irga6 dimerizes in a parallel head-to-head fashion
Source: BMC Biol. 2016 Mar 2;14:14. doi: 10.1186/s12915-016-0236-7 (PMC4774019; doi:10.1186/s12915-016-0236-7)
Supplement: Supplementary file 1 — Supplementary Material (all files combined). Table S1. Data collection statistics. Table S2. Refinement statistics. Figure S1. Mutations R31E, K32E, K176E, K246E eliminate GTP-dependent oligomerization and GTP hydrolysis of Irga6. Figure S2. Packing of Irga6 molecules in the crystal lattice. Figure S3. Contact surfaces in Irga6. Figure S4. Electron density map of the catalytic site in molecule A. (PDF 3.88 mb) [file 12915_2016_236_MOESM1_ESM.pdf]

Additional files

Data collection	data set 1	data set 2	data set 3	merged
Beamline		PETRA III P11		
Wavelength [Å]	0.972	0.972	0.979	
Distance [mm]	1300	850	598	
Space group		$P6_122$		
Cell dimensions				
a, b, c [Å]	98.51	98.51	98.96	98.51
	98.51	98.51	98.96	98.51
	1289.40	1289.40	1.286.21	1289.40
α, β, γ [°]		90, 90, 120		
Resolution [Å]	69.00 (5.80)- 5.48	69.00 (3.16) - 2.99	69.00 (3.06) - 2.90	69.00 (3.20) - 3.00
R_{meas} (%)	10.0 (19.1)	19.5 (97.0)	11.9 (142.7)	22.2 (71.0)
I/σ	24.0 (12.4)	12.0 (1.1)	12.1 (0.8)	10.9 (3.2)
Completeness (%)	99.1 (95.2)	78.1 (37.8)	99.6 (97.5)	100.0 (100.0)
Redundancy	18.3 (13.9)	9.5 (4.2)	7.6 (4.4)	18.4 (6.2)

Table S1: Data collection statistics of three anomalous datasets of the same selenomethionine substituted $Irga6^{R31E,\ K32E,\ K176E,\ K246E}$ crystal measured at different distances. Numbers in parentheses apply to the highest resolution shell.

Refinement	merged
Resolution [Å]	69.0 - 3.2
No. Reflections	64,283
R_{work}[a]/R_{free}[b] [%]	29.7 / 31.7
mol/asu	7
No. protein atoms	22,394
B-factor protein [$Å^2$]	67.5
R.m.s. deviations	
Bond length [Å]	0.003
Angles [°]	0.72

Table S2: Refinement statistics.

[a] $R_{work} = \frac{\sum h,k,l\left||F_{obs}(h,k,l)|-|F_{calc}(h,k,l)|\right|}{\sum h,k,l|F_{obs}(h,k,l|}$.

[b] R_{free} was calculated with 5% of reflections excluded from refinement.

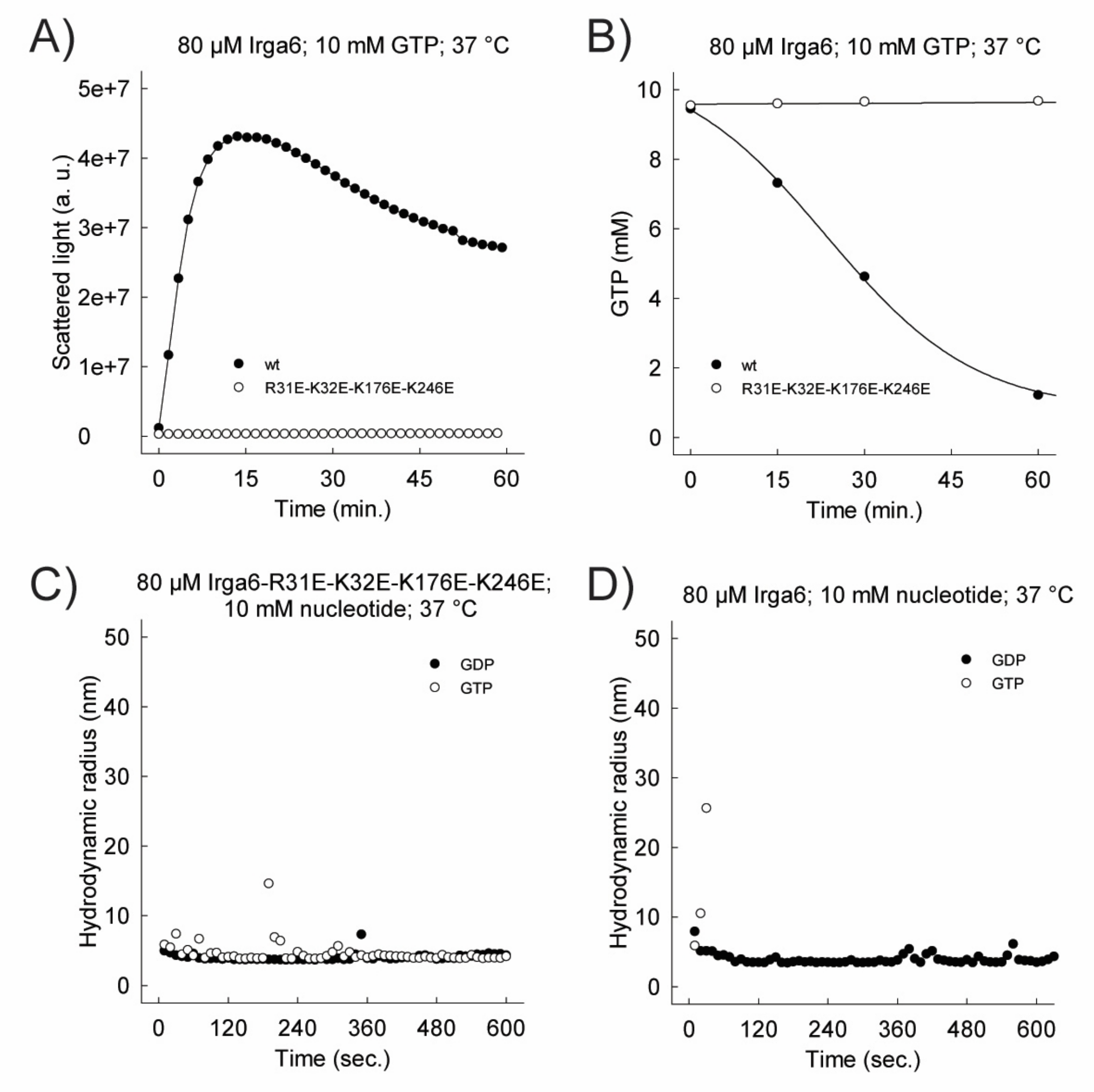

Figure S1: Mutations R31E, K32E, K176E, K246E eliminate GTP-dependent oligomerization and GTP hydrolysis of Irga6.

A) Oligomerization of 80 µM wild-type (WT) or mutant Irga6 proteins was monitored by light scattering in the presence of 10 mM GTP at 37 °C. B) Hydrolysis of 10 mM GTP was measured in the presence of 80 µM WT or mutant Irga6 proteins at 37 °C. Samples were assayed by thin layer chromatography and autoradiography. C) Oligomerization of 80 µM Irga6 mutant proteins was monitored in the presence of 10 mM GDP or GTP by dynamic light scattering (DLS) at 37 °C. D) Oligomerization of 80 µM WT Irga6 proteins was monitored in the presence of 10 mM GDP or GTP by DLS at 37 °C. Note that WT Irga6 in the presence of GTP oligomerized already after 30 sec to such extent that the DLS signal was saturated.

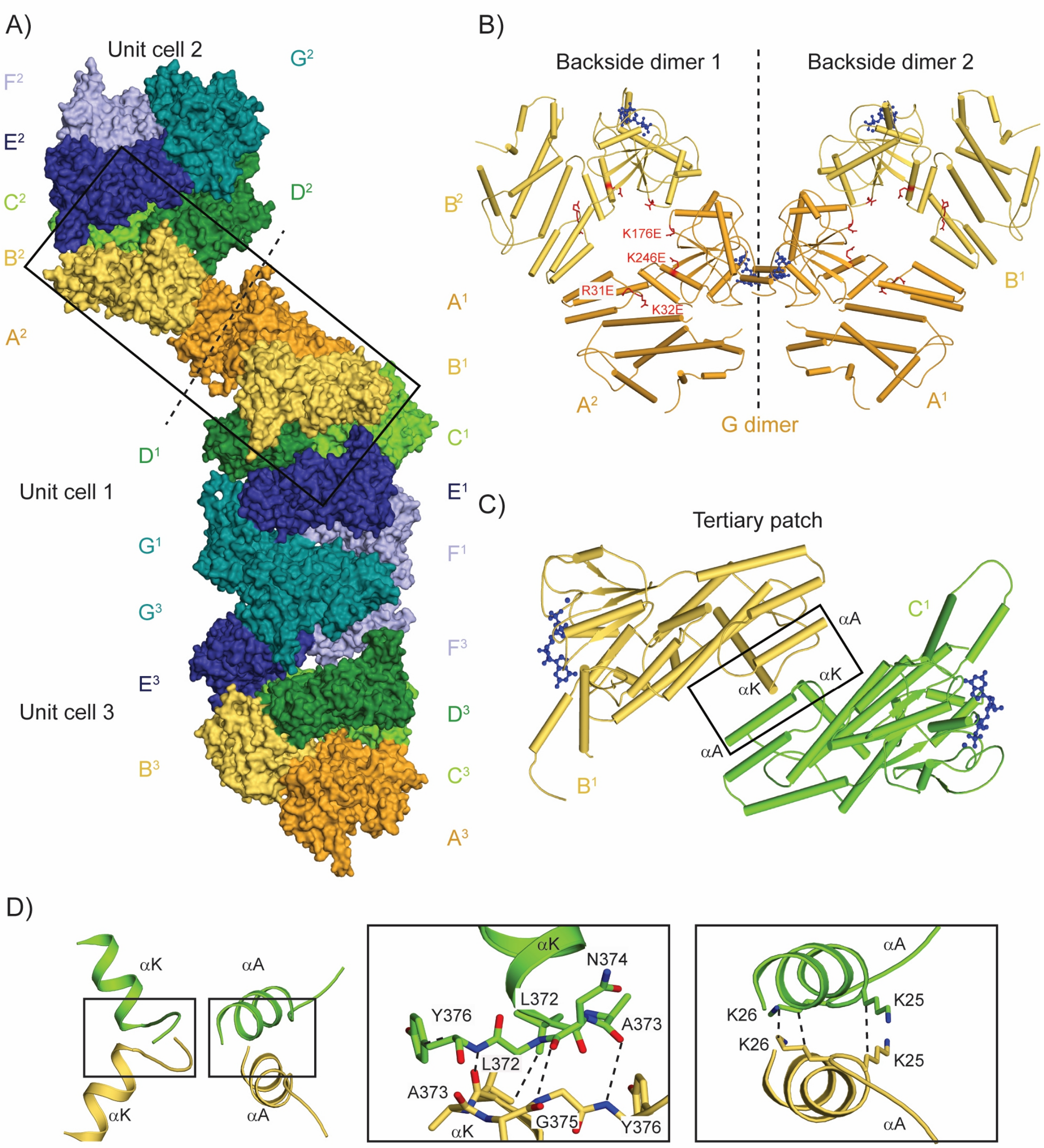

Figure S2: Packing of Irga6 molecules in the crystal lattice.

A) Arrangement of the seven Irga6 molecules A-G in the asymmetric unit and the contacts to the adjacent asymmetric units shown in surface representation. The dotted line indicates a 2-fold axis. B) Backside and G interface dimers (in cylinder representation), as seen in the crystal lattice. Irga6 molecules are color coded as in A. GMPPNP and Mg^{2+} are shown in stick and sphere representation, respectively (blue). The 2-fold axis in the G interface and the mutations introduced in the crystallized constructs are indicated. C) Helices αA and αK of the helical domains establish the tertiary patch. D) Residues involved in the latter interface are shown in stick representation. This contact is observed only between molecules B^1/C^1 and F^1/G^3, whereas molecules D^1/E^1 and A^1/A^4 (not shown here) assemble in a similar orientation, but do not fully engage in these contacts.

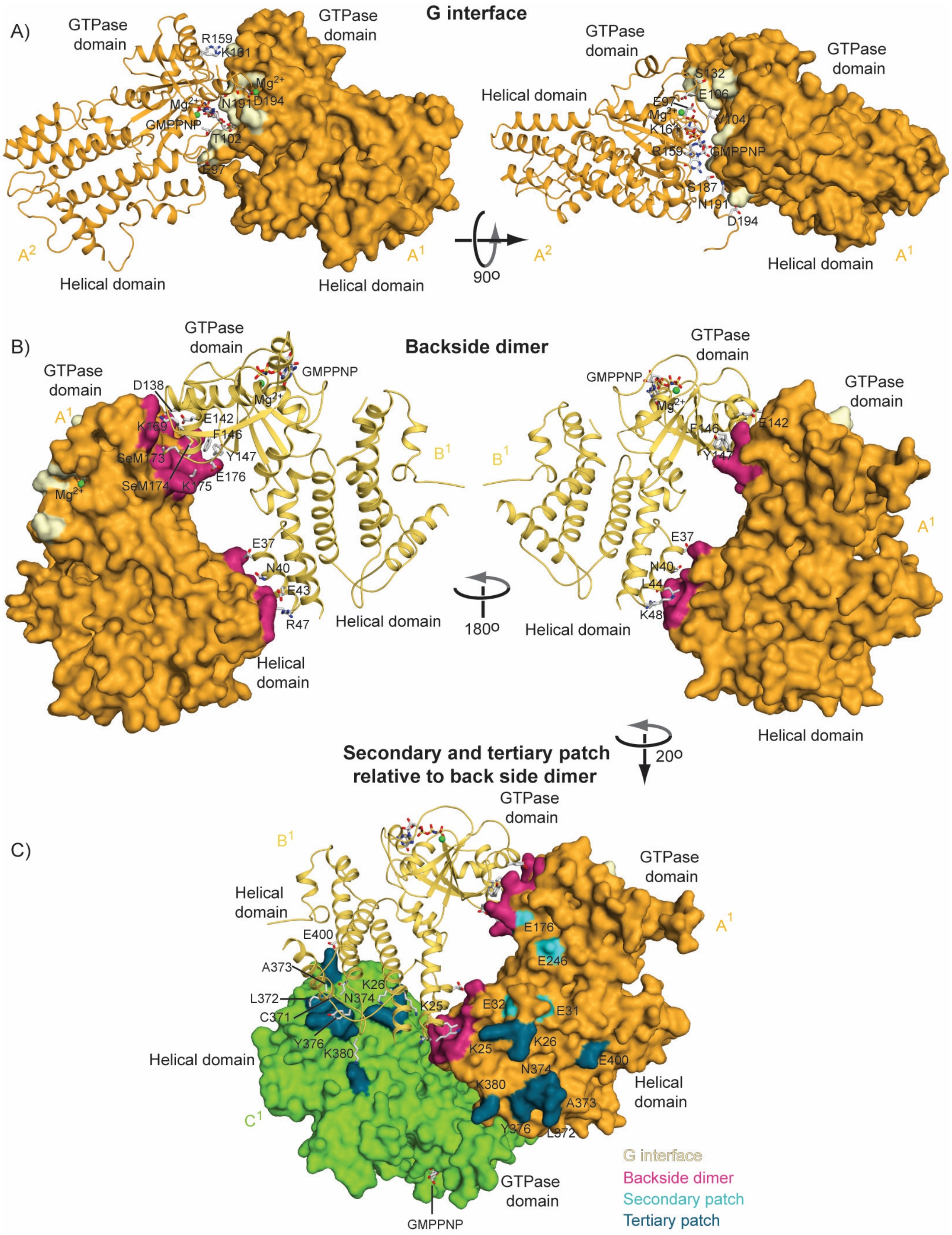

Figure S3: Contact surfaces in Irga6.

Figure S3: Contact surfaces in Irga6.

A) The G interface of Irga6. One molecule is shown in ribbon-type representation and the other in surface representation. Residues in the G interface are shown in stick representation in the left molecule and plotted on the surface of the right molecule (bright yellow). Chains are numbered and colored according to Figure S2.

B) Residues involved in the backside interface are shown in stick representation in one molecule and are plotted in magenta on the Irga6 surface in the other molecule. Note the opposite locations of backside and G interface (plotted in yellow).

C) Residues of the secondary patch (cyan) and tertiary patch (teak) are plotted on the Irga6 surfaces of molecule A^1 and C^1 (for nomenclature, see Figure S2). Residues interacting via the tertiary patch in molecule B^1 (yellow) and C^1 (green) are shown in stick-representation and labelled. Note that the secondary patch is located in between the backside dimer interface and the tertiary patch.

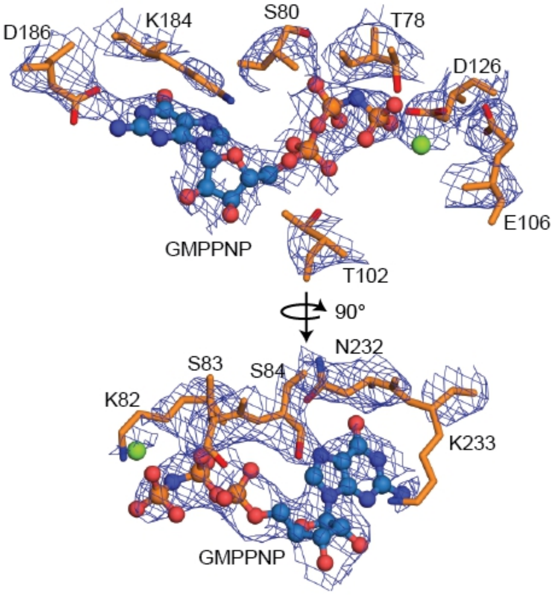

Figure S4: Electron density map of the catalytic site in molecule A.

The electron density (blue) of the refined structure in molecule A was contoured at 1.1 σ. Residues involved in nucleotide binding are labelled and shown in stick representation. GMPPNP and E106 in molecule A show clear electron density. Note that in molecule D, no electron density for GMPPNP was observed and consequently, no nucleotide was included in the model.
